# Update on Vaccine-Derived Polioviruses — Worldwide, July 2012–December 2013

**Published:** 2014-03-21

**Authors:** Ousmane M. Diop, Cara C. Burns, Steven G. Wassilak, Olen M. Kew

**Affiliations:** 1 Department of Immunization, Vaccines, and Biologicals, World Health Organization, Geneva, Switzerland; 2 Division of Viral Diseases, National Center for Immunization and Respiratory Diseases; 3 Global Immunization Division, Center for Global Health, CDC

In 1988, the World Health Assembly resolved to eradicate poliomyelitis worldwide (1). One of the main tools used in polio eradication efforts has been live, attenuated oral poliovirus vaccine (OPV), an inexpensive vaccine easily administered by trained volunteers. OPV might require several doses to induce immunity, but then it provides long-term protection against paralytic disease through durable humoral immunity. Rare cases of vaccine-associated paralytic poliomyelitis can occur among immunologically normal OPV recipients, their contacts, and persons who are immunodeficient. In addition, vaccine-derived polioviruses (VDPVs) can emerge in areas with low OPV coverage to cause polio outbreaks and can replicate for years in persons who have primary, B-cell immunodeficiencies. This report updates previous surveillance summaries (2) and describes VDPVs detected worldwide during July 2012–December 2013. Those include a new circulating VDPV (cVDPV) outbreak identified in Pakistan in 2012, with spread to Afghanistan; an outbreak in Afghanistan previously identified in 2009 that continued into 2013; a new outbreak in Chad that spread to Cameroon, Niger, and northeastern Nigeria; and an outbreak that began in Somalia in 2008 that continued and spread to Kenya in 2013. A large outbreak in Nigeria that was identified in 2005 was nearly stopped by the end of 2013. Additionally, 10 newly identified persons in eight countries were found to excrete immunodeficiency-associated VDPVs (iVDPVs), and VDPVs were found among immunocompetent persons and environmental samples in 13 countries. Because the majority of VDPV isolates are type 2, the World Health Organization has developed a plan for coordinated worldwide replacement of trivalent OPV (tOPV) with bivalent OPV (bOPV; types 1 and 3) by 2016, preceded by introduction of at least 1 dose of inactivated poliovirus vaccine (IPV) containing all three poliovirus serotypes into routine immunization schedules worldwide to ensure high population immunity to all polioviruses (3).

## Properties of VDPVs

Three poliovirus serotypes (PV1, PV2, and PV3) have been identified. Poliovirus isolates are grouped into three categories: 1) WPVs (current WPVs are genetically unrelated to any vaccine strain), 2) vaccine-related polioviruses (VRPVs; <1% divergent [PV1 and PV3] or <0.6% divergent [PV2] from the corresponding OPV strain), and 3) VDPVs (VRPVs >1% divergent [PV1 and PV3] or >0.6% divergent [PV2] from the corresponding OPV strain) (2). VDPVs are further categorized as 1) cVDPVs when evidence of person-to-person transmission in the community exists; 2) iVDPVs, which are isolated from persons with primary, B-cell immunodeficiencies (defects in antibody production); and 3) ambiguous VDPVs (aVDPVs), which are either clinical isolates from persons with no known immunodeficiency and no evidence of transmission or sewage isolates whose source is unknown (2).

VDPVs can cause paralytic polio in humans and have the potential for sustained circulation. VDPVs resemble WPVs biologically (2) and differ from VRPV isolates by having genetic properties consistent with prolonged replication or transmission. Because poliovirus genomes evolve at an overall rate of approximately 1% per year, VRPVs that differ from the corresponding OPV strain by >1% of nucleotide positions (determined by sequencing the genomic region that encodes the major viral surface protein [VP1]) are presumed to have replicated for ≥1 year in one or more persons after administration of an OPV dose and are VDPVs. The typical period of vaccine virus replication is 4–6 weeks in an OPV recipient.

## Virologic Testing for VDPVs

All poliovirus isolates are characterized by laboratories of the Global Polio Laboratory Network (4). The original protocol to screen for VDPVs, using a combination of molecular and antigenic methods, has largely been replaced by a real-time reverse transcription–polymerase chain reaction (rRT-PCR) nucleic acid amplification targeted to nucleotide substitutions that typically revert to the WPV sequence during replication of OPV in the human intestine (5). The rRT-PCR methods have been transferred to 88 of 146 Global Polio Laboratory Network laboratories (4). Candidate VDPVs identified by rRT-PCR screening are sequenced in the VP1 region for definitive analysis; the complete genome is sequenced if required for higher-resolution analysis.

## cVDPVs

**FIGURE 1:**
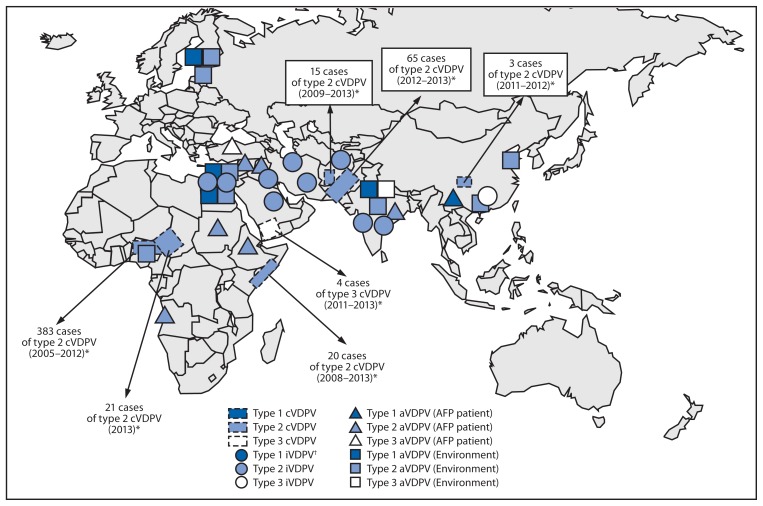
Vaccine-derived polioviruses (VDPVs) detected worldwide — July 2012–December 2013 **Abbreviations:** cVDPV = circulating VDPV; iVDPV = immunodeficiency-associated VDPV; aVDPV = ambiguous VDPV; AFP = acute flaccid paralysis. ^*^Spread of cVDPVs followed the elimination of the corresponding serotype of indigenous wild poliovirus, but with continued introduction of oral poliovirus vaccine into communities with growing immunity gaps. All of the cVDPV outbreaks were detected first by the laboratory, using sequence data and evolutionary analyses. ^†^One type 1 iVDPV case (not shown) identified in an infant in North America (Texas) who had received 2 doses of oral poliovirus vaccine in India.

The number of countries with indigenous cVDPV circulation increased from six to seven since the April 2011–June 2012 reporting period (2). Outbreaks in the Democratic Republic of the Congo (6), Madagascar, Mozambique, and Yemen (type 2 cVDPV [cVDPV2]) appeared to have been interrupted (2); outbreaks identified during the previous period in Afghanistan and Somalia continued; a large outbreak in Nigeria has reached very low incidence (2,7); and new outbreaks were detected in Chad, China, and Yemen. Circulating VDPVs were exported from Chad to Cameroon, Niger, and Nigeria; from Pakistan to Afghanistan; and from Somalia to Kenya. In all countries but Yemen (cVDPV3 outbreak), the cVDPVs detected during this reporting period were type 2 (Table, Figure 1).

### Afghanistan

During July 2009–February 2013, cVDPV2s were isolated from 15 acute flaccid paralysis (AFP) patients and eight contacts from insecure areas of Helmand Province. Two 2012 cVDPV2 isolates from contacts of a child with AFP represented a second emergence in Helmand. Four cVDPV2 isolates from Kandahar Province during October 2012–March 2013 revealed frequent cross-border transmission from Pakistan.

### Cameroon

Among four 2013 cVDPV2 isolates in the Extrême Nord region, two were closely related to isolates from Chad, and two were most closely related to virus circulating in Borno state, Nigeria, and ultimately related to cVDPV2 circulating in Chad.

### Chad

Circulating VDPV2s were isolated from 16 AFP patients during August 2012–May 2013, derived from at least two separate emergences. One (associated with 14 reported cases) apparently originated near N’Djamena and spread eastward within Chad and westward to neighboring parts of Cameroon, Nigeria, and Niger. The other emergence was localized to Ouaddaï region in eastern Chad.

### China

During October 2011–February 2012, cVDPV2s were isolated from three patients with AFP who had received no prior doses of OPV and one contact in Sichuan Province in a county that had gaps in tOPV coverage.

### Kenya

During July 2012–September 2012, three distinct cVDPV2s were isolated from one AFP patient and two contacts in the Dadaab refugee camp near the border with Somalia, representing two separate introductions from the Somalia outbreak.

### Niger

One cVDPV2 was isolated from a patient in Diffa, southeastern Niger, along the border with Nigeria, with onset of AFP in July 2013. The isolate was most closely related to cVDPV2s circulating in Borno state, Nigeria, but appears to have originated in Chad. As with previous cVDPV2 importations (2), no secondary cases were found in Niger.

### Nigeria

The large indigenous cVDPV2 outbreak (383 AFP cases) in northern Nigeria (7), first detected in 2005, appears to have reached very low incidence. During July–December 2012, the indigenous cVDPV2s were isolated from five AFP patients and three contacts. In addition, virus closely related to the indigenous cVDPV2 was isolated from 46 environmental samples. Although the last isolate was from an AFP patient with paralysis onset in December 2012, the indigenous cVDPV2 was detected in 11 environmental samples, most recently in November 2013. During March–November 2013, cVDPV2s were also isolated from five AFP patients and 12 environmental samples in the northeastern states of Borno and Adamawa following importation from Chad.

### Pakistan

During August 2012–December 2013, cVDPV2s were isolated from 61 AFP patients, six contacts, and three environmental samples. The outbreak was associated with an emergence that was first observed in Killa Abdullah, Balochistan Province, with spread in 2013 to the insecure North Waziristan Agency and parts of Karachi in Pakistan, and to Kandahar Province in Afghanistan.

### Somalia

Since 2004, VDPV2s have been isolated from 15 AFP patients and 21 contacts in the southern regions; most were derived from a single emergence. Circulating VDPV2s were isolated from an AFP patient in Lower Juba in July 2012 and from a contact of an AFP case in Lower Shebelle in January 2013.

### Yemen

During April 2012–July 2013, cVDPV3s were isolated from four AFP patients and two contacts in the insecure northwestern governorates of Sa’adah, Hajjah, and Al Hudaydah. The cVDPV3 outbreak followed a cVDPV2 outbreak (11 reported cases, two independent contacts) during April 2011–February 2012.

## iVDPVs

Since the introduction of OPV in 1961, more than 70 persons with primary immunodeficiencies have been found worldwide to be excreting iVDPVs (indicating prolonged infections); the majority of these immunodeficiencies were detected only after onset of AFP. After implementation of intensified surveillance for VDPVs and special studies of iVDPV excretion among persons with primary immunodeficiencies in developing and middle-income countries (8), detection of iVDPV infections increased from two during January 2008–June 2009, to nine during July 2009–June 2011, to 12 during April 2011–June 2012 (2), and declined to 10 during July 2012–December 2013 (Table). Type 2 iVDPVs are the most prevalent (64%), followed by type 1 (21%) and type 3 (15%).

### Afghanistan

A boy aged 36 months with primary immunodeficiency infected with iVDPV2 developed AFP in October 2013.

### China

A boy aged 7 months with primary immunodeficiency was infected with iVDPV3 after receiving three tOPV doses and developed AFP in May 2013.

### Egypt

Two infants, aged 6 months and 5 months, with severe combined immunodeficiency who did not have AFP were found to be infected with iVDPV2s in July 2012 and November 2013, respectively.

### India

A girl aged 4 months with hypogammaglobulinemia and infected with iVDPV2 developed AFP in 2013, and a child with agammaglobulinemia infected with iVDPV2 developed AFP and died in 2013.

### Iran

Iran has maintained sensitive clinical and laboratory surveillance to screen persons with primary immunodeficiencies for poliovirus infections. During this reporting period, two AFP patients were found to be excreting iVDPVs. A boy aged 11 months with primary immunodeficiency and infected with iVDPV2 developed AFP in August 2012, and a boy aged 13 months with primary immunodeficiency infected with iVDPV2 developed AFP in January 2013.

### Iraq

A boy aged 24 months with primary immunodeficiency infected with iVDPV2 developed AFP in July 2012 and died in December 2013.

### Saudi Arabia

A girl aged 2 years was taken to Germany for treatment for severe combined immunodeficiency. She did not have AFP, but was found to be infected with iVDPV2.

### United States

A boy aged 7 months with severe combined immunodeficiency who had received 2 doses of OPV in India was infected with iVDPV1 and developed AFP in July 2013, 2 weeks after arrival in the United States; he died 3 weeks after symptom onset.

## aVDPVs

During June 2012–December 2012, aVDPVs were isolated in 13 countries (Table). The most divergent aVDPVs were continuations of lineages previously detected in sewage samples in Estonia, Finland, and Israel, countries with >90% polio vaccination coverage. The persons infected with the corresponding aVDPVs have not been identified. Detection of aVDPVs in settings (including local pockets) with <60% polio vaccination coverage might signal cVDPV emergence and potential gaps in surveillance. Some aVDPVs, especially those with limited divergence in areas with high vaccination coverage and in patients with no known immunodeficiency, might represent limited spread of OPV virus or the upper limit of OPV sequence divergence in a single normal vaccine recipient or contact.

### Angola

An AFP patient with no known immunodeficiency was infected with aVDPV2 in October 2013.

### China

A boy aged 18 months with no known immunodeficiency who received 2 OPV doses in Myanmar traveled to Yunnan, China, after AFP onset and was found to be infected with aVDPV1. Environmental aVDPV2 isolates were detected in Shandong and Guangdong provinces.

### Egypt

An aVDPV1 was isolated from Alexandria sewage collected in 2012, and unrelated aVDPV2s were isolated from sewage collected in nine sites in eight cities during 2012–2013.

### Estonia

Two aVDPV2s, closely related to isolates detected in 2008–2010 in Estonia, were detected in Tallinn sewage collected in December 2012. Shared noncapsid sequences with a similarly divergent aVDPV3 strongly suggest origination from a chronic iVDPV excretor (9).

### Ethiopia

An aVDPV2 was isolated in July 2012 from an AFP patient with no known immunodeficiency.

### Finland

Highly divergent aVDPV1s and aVDPV2s were isolated from sewage samples collected in Tampere in 2013. The aVDPV isolates were unrelated to aVDPVs found in Estonia, but were closely related to sewage isolates detected during 2008–2012 in Finland, and were likely derived from a single tOPV dose (2).

### India

In 2013, an aVDPV2 was isolated from an AFP patient with no known immunodeficiency. In addition, one aVDPV1, nine aVDPV2s, and one aVDPV3 were isolated from sewage samples during the reporting period.

### Iraq

A boy aged 24 months with no known immunodeficiency infected with VDPV2 developed AFP in November 2012.

### Israel

Highly divergent aVDPV2s had been detected in sewage samples in the Tel Aviv area since 1998 (2), and highly divergent aVDPV1s had been detected in sewage samples in Haifa since 2009. The aVDPVs appear to be derived from different chronic excretors.

### Nigeria

Environmental isolates closely related to cVDPVs known to be circulating in Nigeria were classified as cVDPVs. Three aVDPV2s were isolated from AFP patients during March–October 2013, and two aVDPV2s were isolated from sewage samples collected at two sites in Sokoto state in June 2012 and January 2013.

### Sudan

An aVDPV2 was isolated from a contact of an AFP patient in February 2013.

### Syria

An aVDPV2 was isolated from an AFP patient with no known immunodeficiency in November 2012.

### Turkey

An aVDPV3 was isolated from a contact of an AFP patient in September 2012.

### Discussion

Circulating VDPV outbreaks continue to emerge in settings of conflict and insecurity, poor infrastructure, and widening immunity gaps. Outbreaks in Afghanistan, Nigeria, Pakistan, and Somalia have occurred in areas with recent WPV circulation and where conflict and insecurity has limited access of immunization teams to children. As with WPVs, cVDPVs can spread to neighboring countries, causing sporadic cases and outbreaks. In Afghanistan and Nigeria, polio cases were associated with indigenous and imported cVDPV2s. When children are accessible, cVDPV outbreaks have been stopped by supplementary immunization activities (SIAs).* The large and prolonged indigenous cVDPV2 outbreak in northern Nigeria appears to have reached very low incidence by successive tOPV SIA rounds of increasing quality, but a new outbreak from imported cVDPV2 occurred in insecure areas of the northeast.

**FIGURE 2:**
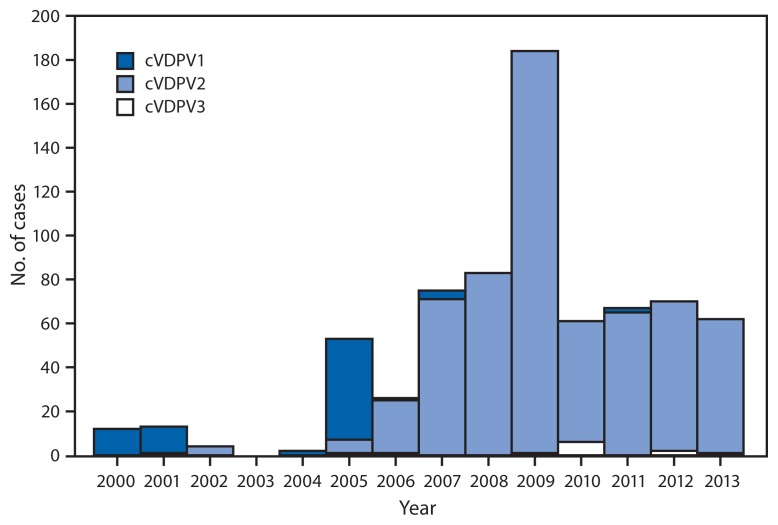
Circulating vaccine-derived poliovirus (cVDPV) cases detected worldwide, by serotype and year — 2000–2013

Since eradication of WPV2 in 1999, all poliomyelitis cases associated with PV2 have resulted from the continued use of tOPV. Moreover, the serotype profile of cVDPVs has shifted in recent years, with cVDPV2s representing 13.1% of the 84 cVDPV cases reported during 2000–2005, and 97.1% of the 628 cVDPV cases reported during 2006–2013 (Figure 2). In view of the rising incidence of cVDPV2 outbreaks, the Global Polio Eradication Initiative has incorporated coordinated worldwide withdrawal of tOPV and replacement with bOPV into its new strategic plan, ultimately leading to withdrawal of all OPV use (3). The switch from tOPV to bOPV, planned for April 2016, is predicated on the complete cessation of cVDPV2 transmission and will require intensification of AFP and poliovirus surveillance. Routine immunization will be strengthened, and in countries using OPV, 1 dose of IPV will be given with the third dose of diphtheria-pertussis-tetanus vaccine. Large stockpiles of monovalent OPV will be maintained, and a robust surveillance and response capacity will be established (3).

What is already known on this topic?

Genetically divergent vaccine-derived polioviruses (VDPVs) are detected by poliovirus surveillance and have biologic properties indistinguishable from wild polioviruses. High poliovirus vaccination coverage can prevent circulating VDPV (cVDPV) outbreaks, but prolonged immunodeficiency-associated VDPV (iVDPV) infections will occur as long as oral poliovirus vaccine (OPV) is used.

What is added by this report?

Although recent cVDPV outbreaks in two countries have apparently stopped, and the large outbreak in Nigeria has nearly stopped, outbreaks continue in Afghanistan and Somalia, and new outbreaks have been detected in Chad, Pakistan, and Yemen. Ten new prolonged iVDPV infections in eight countries were detected, with increasing numbers found in developing and middle-income countries. Since 2006, >97% of cVDPVs are type 2.

What are the implications for public health practice?

Circulating VDPV outbreaks can be prevented and controlled by high OPV coverage; however, only cessation of OPV use will prevent prolonged iVDPV infections. To address the continued global type 2 VDPV risk, the World Health Organization recommends 1) shifting from trivalent OPV (tOPV) to bivalent OPV (types 1 and 3) by April 2016, 2) including at least 1 dose of inactivated poliovirus vaccine into routine immunization schedules worldwide, 3) maintaining strategic stockpiles of monovalent OPV, 4) developing a robust acute flaccid paralysis and poliovirus surveillance and response capacity, and 5) encouraging development of antiviral drugs to clear prolonged iVDPV infections.

Replacement of tOPV with bOPV will greatly reduce the risk for cVDPV2 outbreaks, and global cessation of OPV use (3) will prevent virtually all cVDPV outbreaks and all new iVDPV infections. However, a small number of persons with chronic iVDPV infections are likely to continue to excrete poliovirus for at least a decade after the administration of the last OPV dose. Therefore, maintenance of high levels of population immunity by comprehensive coverage with IPV will be essential to protect against possible iVDPV spread in the community. Detection of chronic iVDPV excretors in all countries (8) and clearing their infections also will be important (10).

## Figures and Tables

**TABLE:** Vaccine-derived polioviruses (VDPVs) detected worldwide — July 2012–December 2013

Category	Country	Year(s) detected*	Source (total cases or specimens)†	Sero-type	No. of isolates§ July 2012–December 2013			VP1 divergence from Sabin OPV strain (%)	Routine coverage with 3 doses of polio vaccine (%)¶	Estimated duration of VDPV replication (yrs)**	Current status (date of last outbreak case, last patient isolate, or last environmental sample)

					Cases	Contacts	Non-AFP Source				
**cVDPV** ††	Afghanistan	2009–2013	Outbreak (15 cases)	2	8	8	—	(0.9–5.5)	(71)	5	February 13, 2013
	Afghanistan	2012–2013	Importation§§	2	4	—	—	(2.0–2.3)	(71)	—	March 13, 2013
	Cameroon	2013	Importation¶¶	2	4	—	—	(1.2–2.0)	(85)	—	August 12, 2013
	Chad	2012–2013	Outbreak (16 cases)	2	16	—	—	(0.7–1.8)	(56)	1.5	May 12, 2013
	China	2011–2012	Outbreak (3 cases)	2	3	1	—	(0.7–1.8)	(99)	1.5	February 8, 2012
	Kenya	2012	Importation***	2	1	2	—	(4.3–4.9)	(82)	—	August 29, 2012
	Niger	2013	Importation†††	2	1	—	—	(2.1)	(78)	—	July 14, 2013
	Nigeria§§§	2005–2013	Outbreak (383 cases)¶¶¶	2	12	1	57	(0.7–7.3)	(59)	9	November 18, 2013
	Nigeria	2013	Importation¶¶	2	4	—	12	(1.2–2.4)	(59)	—	November 20, 2013
	Pakistan	2012–2013	Outbreak (61 cases)	2	61	6	3	(0.7–3.3)	(75)	3	December 30, 2013
	Somalia	2008–2013	Outbreak (19 cases)	2	1	1	—	(3.3–4.0)	(47)	5	January 9, 2013
	Yemen	2011–2013	Outbreak (4 cases)	3	4	2	—	(2.0–3.0)	(89)	2.5	July 25, 2013
**iVDPV**	Afghanistan	2013	AFP patient PID	2	1	—	—	(0.9)	(71)	<1	October 22, 2013
	China	2013	AFP patient PID	3	1	—	—	(1.3)	(99)	<1	May 19, 2013
	Egypt	2012	Non-AFP patient PID	2	1	—	—	(1.1)	(99)	~1	July 4, 2012
	Egypt	2012	Non-AFP patient PID	2	1	—	—	(1.0)	(99)	<1	November 11, 2012
	India	2013	AFP patient AGG	2	1	—	—	(0.9)	(70)	<1	2013
	Iran	2012	AFP patient PID	2	1	—	—	(1.1)	(99)	<1	August 12, 2012
	Iran	2013	AFP patient PID	2	1	—	—	(0.9)	(99)	<1	January 10, 2013
	Iraq	2012	AFP patient PID	2	1	—	—	(1.0)	(70)	<1	July 11, 2012
	Saudi Arabia	2013	Non-AFP patient SCID	2	—	—	—	(4.4)	(98)	4	2013
	United States	2013	AFP patient SCID	1	1	—	—	(1.3)	(93)	<1	July 7, 2013
**aVDPV**	Angola	2013	AFP patient	2	1	—	—	(0.8)	(88)	<1	October 6, 2013
	China****	2012	AFP patient	1	1	—	—	(2.3)	(99)	~2	May 29, 2012
	China	2013	AFP patient	3	1	—	—	(1.3)	(99)	~1	May 19, 2013
	China	2012–2013	Environment	2	—	—	2	(0.7–0.8)	(99)	<1	June 7, 2013
	Egypt	2012–2013	Environment	1	—	—	1	(1.1)	(99)	~1	2012
			Environment	2	—	—	9	(0.7–1.8)			April 14, 2013
	Estonia	2008–2012	Environment	2	—	—	2	(16.2)	(94)	>15	December 27, 2012
	Ethiopia	2012	AFP patient	2	1	—	—	(0.9)	(70)	<1	July 20, 2012
	Finland	2008–2013	Environment	1	—	—	6	(12.9–14.0)	(99) (IPV)	>15	December 9, 2013
		2008–2013	Environment	2	—	—	2	(15.5)		>15	May 13, 2013
	India	2013	AFP patient	2	1	—	—	(0.7)	(70)	<1	2013
	India	2012–2013	Environment	1	—	—	1	(1.0)	(70)	~1	2012
			Environment	2	—	—	9	(0.7–1.4)		≤1	2013
			Environment	3	—	—	1	(1.0)		~1	2013
	Iraq	2012	AFP patient	2	1	—	—	(0.9)	(70)	<1	November 10, 2012
	Israel	2009–2012	Environment	1	—	—	1	(13.8)	(95)	>10	December 18, 2012
		1998–2013	Environment	2	—	—	4	(16.3)		>15	May 29, 2013
	Nigeria	2012–2013	AFP patients	2	4	—	—	(0.7–0.8)	(57)	<1	October 28, 2013
			Environment	2	—	—	2	(0.7)		<1	January 21, 2013
	Sudan	2013	AFP contact	2	—	1	—	(0.7)	(93)	<1	February 24, 2013
	Syria	2012	AFP patient	2	1	—	—	(1.3)	(52)	~1	November 22, 2012
	Turkey	2012	AFP contact	3	—	1	—	(1.1)	(97)	~1	September 23, 2012

**Abbreviations:** cVDPV = circulating VDPV; iVDPV = immunodeficiency-associated VDPV; aVDPV = ambiguous VDPV; OPV = oral poliovirus vaccine; IPV = inactivated poliovirus vaccine; AFP = acute flaccid paralysis; AGG = agammaglobulinemia; PID = primary immunodeficiency; SCID = severe combined immunodeficiency.

* Total years detected and cumulative totals for previously reported cVDPV outbreaks (Nigeria and Somalia).

† Outbreaks list total cVDPV cases. Some VDPV case isolates from outbreak periods might be listed as aVDPVs.

§ Total cases for VDPV-positive specimens from AFP cases and total VDPV-positive samples for environmental (sewage) samples.

¶ Based on 2012 data from the World Health Organization (WHO) Vaccine Preventable Diseases Monitoring System (2013 global summary) and WHO-United Nations Children’s Fund (UNICEF) coverage estimates, available at http://www.who.int/immunization_monitoring/en/globalsummary/countryprofileselect.cfm. National data might not reflect weaknesses at subnational levels.

** Duration of cVDPV circulation was estimated from extent of VP1 nucleotide divergence from the corresponding Sabin OPV strain; duration of immunodeficiency-associated VDPV replication was estimated from clinical record by assuming that exposure was from initial receipt of OPV; duration of ambiguous VDPV replication was estimated from sequence data.

†† All cVDPV isolates except those from China were vaccine/nonvaccine recombinants.

§§ Importation from Pakistan.

¶¶ Importation from Chad.

*** Importation from Somalia.

††† Importation from Nigeria of cVDPV2 originating in Chad.

§§§ All Nigerian cVDPV2 isolates in 2012 from AFP patients, contacts, and the environment were indigenous.

¶¶¶ Count does not include 29 cases with <10 substitutions in VP1 detected before 2010.

**** Patient from Myanmar.

## Footnotes

* SIAs are mass vaccination campaigns conducted in a short period (days to weeks) during which a dose of OPV is administered to all children aged <5 years, regardless of previous vaccination history. Campaigns can be conducted nationwide or in portions of a country.
